# Colorectal Carcinoma with Extremely Low CA19-9

**DOI:** 10.1155/2009/780263

**Published:** 2009-08-24

**Authors:** Yutaka J. Kawamura, Aika Tokumitsu, Junichi Sasaki, Shingo Tsujinaka, Takafumi Maeda, Ken Mizogami, Fumio Konishi

**Affiliations:** Department of Surgery, Saitama Medical Center, Jichi Medical University, 1-847 Amanuma-cho, Omiya-ku, Saitama 330-8503, Japan

## Abstract

*Aim*. The aim of this study is to determine the significance of postoperative sequential measurements of serum CA19-9 in patients with extremely low serum level. *Patients and Methods*. Serum level of CA19-9 of 1096 patients who underwent surgery was measured preoperatively and every three months after surgery for 5 years. Patients with CA19-9 level of less than 2 U/mL at the time of diagnosis were defined as Extremely Low CA19-9 (ELCA). *Results*. One hundred and seven patients (9.8%) were ELCA. Of these, 86 underwent surgery with curative intent. Serum levels of CA19-9 in patients who did not undergo curative resection (*N* = 12) and who developed recurrence (*N* = 10) were less than 2.0 U/mL in all occasions during followup. In all patients without recurrence, serum level of CA19-9 also remained less than 2.0 U/mL. *Conclusion*. In patients with extremely low CA19-9, who consist of 9.8% of colorectal carcinoma cases, postoperative sequential measurement of serum level of CA19-9 contributed neither to assessment of curability of surgical resection nor to detection of recurrence.

## 1. Introduction

For patients with colorectal carcinoma, assessment of curability of the surgery as well as that of recurrence is a very important issue. In fact, the close follow up program aiming at early detection of the recurrence after potentially curative surgery has been reported to improve survival rate [1–6]. Although the “best” program for detection of the recurrence and for the treatment failure is not yet determined, the combination of two modalities such as imaging studies and tumor markers is generally used.

Among tumor markers used in the field of gastroenterology, carcinoembryonic antigen (CEA) has been recommended in several guidelines and widely used for postoperative follow up as well as for the assessment of efficacy of the treatment [7–9]. CA19-9 has also been used as a tumor marker for colorectal carcinoma [10–13], usually accompanied with CEA [14–17].**

CA19-9 is an antibody to carbohydrate chain sialyl Lewis a (sLe^a^) which is synthesized by gylcosyltransferases [18], and it is well known that in certain group of patients serum level of CA19-9 is very low [19, 20]. However, little is known about the serum level of CA19-9 in these patients with special regards to progression and recurrence of colorectal carcinoma. The aim of this study is to clarify the changes of serum level of CA19-9 in these patients and elucidate its clinical significance.

## 2. Materials and Methods

Patients with colorectal carcinoma who were treated from 2000 to 2005 in our department were studied. Serum level of CA19-9 was measured preoperatively and every three month for 5 years postoperatively. If postoperative serum CA19-9 was elevated above the normal limit, the measurement was done after one month. And if elevation continued, imaging study was performed. In this study, the recurrence was diagnosed by the imaging studies, not solely by the elevation of tumor marker. Serum CA19-9 was measured by chemiluminescence enzyme immunoassay method. The upper limit of normal range was 37 U/mL, and the lowest measuring limit was 2 U/mL. Patients with CA19-9 level of less than 2 U/mL at the time of diagnosis of colorectal carcinoma were clinically defined as Extremely Low CA19-9 (ELCA). The change of serum CA19-9 level in these patients was analyzed with special regards to recurrence of colorectal carcinoma.

This study was approved by the Institutional Review Board. Informed consent from each patient was exempted by the Institutional Review Board of Jichi Medical University due to retrospective nature of this study.

## 3. Results

From 2000 to 2007, 1096 patients with colorectal carcinoma were treated. Of these patients, 107 (9.8%) were ELCA. There were 65 males and 42 females with mean age of 64.0 (range, 29–86). Mean follow up period was 3.8 years (range: 1.0 to 5.3 years), and serum CA19-9 was measured 1626 times for these 107 patients. Numbers of patients with carcinoma in the cecum, ascending colon, transverse colon, descending colon, sigmoid colon, and rectum were 8, 19, 5, 2, 31, and 42, respectively. Numbers of patients with clinical stages I, II, III, and IV were 31, 32, 27, and 17, respectively (Table 1). 

**Figure 1:**
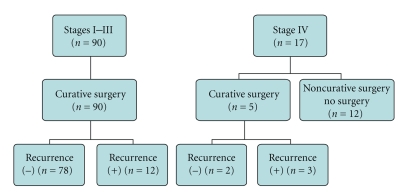
Schematic representation of clinical course of clinical CA19-9 cases.

Clinical course of these patients was represented in Figure 1. Of 17 patients with stage IV disease, five patients did not undergo surgery, and seven patients underwent resection with noncurative intent. These 12 patients remained to be clinically CA19-9 negative during follow up. 

All patients with stages I, II, and III and five patients with stage IV disease underwent potentially curative surgery, and 15 patients developed recurrence. Serum levels of CA19-9 were less than 2.0 U/mL during follow up in patients both with and without recurrence.

## 4. Discussion

Early detection of the recurrence after potentially curative surgery is an important issue in management of the patients with colorectal carcinoma [1–6]. Several studies revealed improved survival by intensive postoperative follow up program. Although there are differences in follow up programs among the studies, the imaging study and the measurement of serum tumor marker are included in postoperative follow up. 

Measurement of tumor marker is also used in the assessment of efficacy of chemotherapy, especially in those without measureable disease [21, 22].

sLe^a^, which is recognized by CA19-9, is a carbohydrate chain which is expressed on tumor cells and is known to play a role in adhesion between tumor cells and endothelial cells [18]. sLe^a^ is secreted into the serum and is used as a tumor marker for pancreatic, hepatobiliary, gynecological, and colorectal carcinoma [9, 23–25]. Several studies revealed the usefulness of the measurement of serum CA19-9 [10–15, 26–29]; however, some studies failed to show its usefulness [30–34], and measurement of CA19-9 has not been recommended in guidelines [7–9]. Main focus of the previous studies was its accuracy in terms of predicting prognosis and its relevance to recurrence, though, only a few studies paid attention for patients with low serum CA19-9 value [19, 20]. 

In this study, we analyzed the serum level of CA19-9 in patients with colorectal carcinoma in order to clarify the changing pattern in serum CA19-9 and to determine the clinical role of its measurement. CA19-9 is an antigen which recognizes carbohydrate chain sLe^a^, and the value of CA19-9 means the amount of the carbohydrate chains in sera, which is called secreted type sLe^a^. The amount of secreted type sLe^a^ is determined by phenotype of both the Lewis (Le) and secretor (Se) genes. Narimatsu studied the correlation between phenotype of these two genes and the amount of serum sLe^a^ and found that there is no secretion of sLe^a^ in patients with phenotype of le/le and se/se. However, there are only a few studies concerning patients with low serum CA19-9 level. In this study, we defined ELCA as having lower than measuring limit and studied the relation of clinical course and serum level of CA19-9 [19, 20].

Although the synthesis of CA19-9 increases in a certain group of patients with colorectal carcinoma, particularly those with advanced disease, this study demonstrated that serum level of CA19-9 remains lower than the measurable limit in all ELCA cases irrespective of the stage at the time of diagnosis, progression, and recurrence. Our clinical definition of ELCA may include not only genetic Lewis negative cases but also the case with very weak expression of CA19-9; however, with our broad definition of Lewis negativity, our study showed no benefit of serial measurement of serum CA19-9 in patients with colorectal carcinoma whose serum level of CA19-9 was lower than measurable limit at the initial assessment.

Further investigation is necessary to elucidate the significance of CA19-9 in entire patients with colorectal carcinoma; however, we found that our criterion of ELCA is practically useful to determine the group of patients for whom serial measurement of serum CA19-9 is not contributory for clinical management. Additionally, because the previous studies focusing on the clinical significance of CA19-9 included patients who do not secrete CA19-9, who consisted of 9.8% of the patients with colorectal carcinoma, the exclusion of these patients may provide more precise information concerning the clinical significance of serum CA19-9. 

We conclude that the measurement of serum CA19-9 should be omitted irrespective of clinical setting if the result of the first measurement of serum CA19-9 was less than lower limit. Because CA19-9 was used in variety of adenocaricnoma, we believe that further investigation concerning the clinical significance of CA19-9 in patients with other organ malignancies with less than measuring limit may facilitate the saving of medical cost.

## Figures and Tables

**Table 1:** Clinical characteristics of clinical CA19-9 negative cases (*n* = 75).

Age	64.0 (29–86)
Gender (M/F)	65/42
Location	
* *Cecum	8
* *Ascending	19
* *Transverse	5
* *Descending	2
* *Sigmoid	31
* *Rectum	42
TNM stage	
* *I	31
* *II	32
* *III	27
* *IV	17
